# Exploring Insights Into Vaginal Microbiome Profiles in Relation to Intrauterine Insemination Success Rates

**DOI:** 10.7759/cureus.92244

**Published:** 2025-09-13

**Authors:** Mamoona Kouser, Shama Chaudhry, Maryum Sana

**Affiliations:** 1 Obstetrics and Gynecology, Pakistan Air Force Hospital, Islamabad, PAK; 2 Obstetrics and Gynecology, Ziauddin University, Karachi, PAK; 3 Nursing and Patient Care, University of Health Sciences, Lahore, PAK

**Keywords:** artificial, assisted, fertility, insemination, lactobacillus, microbiota, reproductive techniques

## Abstract

Background: The vaginal microbiome has an essential role in female reproduction, especially during assisted reproduction treatments. The objective of the study was to examine vaginal microbiome profiles of women following intrauterine insemination (IUI) procedures and determine how they relate to clinical pregnancy outcomes.

Materials and methods: A prospective observational study was conducted on 100 women with IUI treatment. Before the procedure, vaginal swabs were taken and sequenced using the 16S rRNA gene to determine microbial community types. Based on microbiome profiles, patients were divided into *Lactobacillus*-dominant (n = 68) and non-*Lactobacillus*-dominant (n = 32) groups. Clinical pregnancy rates were compared between the two groups. SPSS Statistics version 26.0 (IBM Corp. Released 2019. IBM SPSS Statistics for Windows, Version 26.0. Armonk, NY: IBM Corp.) was used, with p < 0.05 considered statistically significant.

Results: Overall, 68% of the participants had *Lactobacillus*-dominant vaginal microbiomes, whereas 32% had varied non-*Lactobacillus* profiles. The rate of clinical pregnancy was significantly improved in the *Lactobacillus*-dominant group compared to the non-dominant group, 26 (38.2%) vs. 4 (12.5%), p = 0.008. Logistic regression analysis revealed that the *Lactobacillus*-dominant profile was independently associated with the success of IUI (adjusted OR 3.85; 95% CI 1.28-11.58; p = 0.016). There were no significant differences in age, body mass index, and infertility duration between the two groups.

Conclusions: The success rates of IUI were significantly associated with vaginal microbiome composition, particularly the predominance of *Lactobacillus*. These results support that microbiome profiling may become a helpful technique to predict and enhance the success rates in fertility treatment.

## Introduction

Generally, 8-16% of all couples in the reproductive age are infertile, with roughly 30% showing no identifiable cause even after a comprehensive clinical investigation [1]. The importance of the vaginal microbiome in female reproductive health has been a growing interest in recent years [2]. The preservation of beneficial *Lactobacillus* species, primarily *L. crispatus* and *L. gasseri*, is linked to improved fertility due to their ability to maintain a vaginal environment with acidic pH and suppression of pathogens [3]. In contrast, low *Lactobacillus* abundance or high anaerobic diversity has been associated with poor reproductive outcomes [4]. A *Lactobacillus*-associated vaginal and endometrial flora is associated with increased implantation and clinical pregnancy rates in assisted reproductive technology (ART) procedures, especially in vitro fertilization (IVF) [5].

Compared to IVF, intrauterine insemination (IUI) is less invasive, cheaper, and achieves a success rate of 10-20% per cycle [6]. Nevertheless, the role of the vaginal microbiome in IUI outcomes is understudied despite its frequent usage. Significant research has been conducted on ART, resulting in a lack of knowledge about the vaginal microbiome’s impact on IUI success. Moreover, although microbiome-based diagnostic technologies constitute a significant focus, their actual implementation in IUI situations is not yet validated [7]. Knowledge of this relationship is crucial for the optimization of noninvasive fertility treatments. Profiling of the microbiome may inform personalized therapeutic approaches before treatment, such as probiotics or antimicrobial manipulation to increase success [8].

The objective of this study was to determine the correlation of vaginal microbiome with clinical pregnancy after IUI. The aim was also to assess the extent of the relationship represented by the prevalence of *Lactobacillus*. Furthermore, this study evaluated the possible applicability of microbiome profiling in IUI treatment planning.

## Materials and methods

Study design, setting, duration, and ethical considerations

This prospective observational study aimed to investigate the association between vaginal microbiome profile and clinical pregnancy outcomes of women receiving IUI treatment. The study was conducted at the Department of Obstetrics and Gynecology in a tertiary care setting from March 2023 to August 2023, after obtaining informed consent from the Shaikha Fatima Institute of Nursing and Health Sciences (approval number: MLS/23/1435).

Sampling technique and sample size calculation

Consecutive non-probability sampling was used to recruit 100 women aged 20-38 years who underwent IUI. The OpenEpi version 3.0.0 (released in 2013, Atlanta, GA, USA) was used to calculate the sample size based on a 65% prevalence of *Lactobacillus* in fertile women, a 95% confidence level, 80% power, and a 10% margin of error [9].

Inclusion and exclusion criteria

The women between 20 and 38 years old who underwent IUI with primary or secondary infertility, regular ovulatory cycles, bilateral tubal patency confirmed by hysterosalpingography or laparoscopy, and normal uterine anatomy confirmed by ultrasound or hysteroscopy were included in the study. Active genital infection, recent antibiotic or probiotic use (within the last 30 days), systemic diseases (uncontrolled diabetes, autoimmune disorders), and severe endometriosis/uterine abnormalities (large fibroids, adhesions) were exclusion criteria. Any patients with recurrent pregnancy loss (≥ 3 consecutive losses) or patients participating in another clinical trial were also excluded.

Group allocation, data collection, and microbiome analysis

The participants were divided into two groups based on vaginal microbiome: *Lactobacillus*-dominant (n = 68) and non-*Lactobacillus*-dominant (n = 32). Aseptically collected vaginal swabs were obtained before intervention, i.e., ovulation induction and any medication or insemination procedure. Microbial DNA was extracted using a commercially available QIAamp DNA mini kit (Qiagen, Germany) with an extra step for the gram-positive cell wall lysis (mechanical). Bacterial profiling was performed by 16S rRNA gene sequencing. Compliance was achieved by acquiring a pre-intervention sample with medication or insemination. A validated microbial sequencing platform, such as the Illumina MiSeq (USA), was used to profile the microbiome, and taxonomic classification was performed using the SILVA database [10]. Ultrasound confirmation of an intrauterine gestational sac at six to seven weeks was used to confirm clinical pregnancy.

Statistical software, test, and significance value

SPSS Statistics version 26.0 (IBM Corp. Released 2019. IBM SPSS Statistics for Windows, Version 26.0. Armonk, NY: IBM Corp.) was used to analyze data. Categorical variables were expressed as frequencies and percentages and analyzed using the chi-square test. Continuous variables were expressed as mean ± standard deviation using the independent t-test. Multivariable logistic regression was performed to assess independent predictors of IUI success, with adjusted odds ratios (OR) and 95% confidence intervals (CI). The p-value < 0.05 was considered statistically significant.

## Results

This research aimed to evaluate the effects of vaginal microbiome composition on the success of IUI in 100 women. Out of these, 68 (68%) were *Lactobacillus*-dominant and 32 (32%) were non-*Lactobacillus*-dominant. Clinical pregnancy was more likely to be present in the *Lactobacillus*-dominant group compared to the non-dominant group. Dominance of *Lactobacillus* was also found to be an independent predictor of IUI success. Age differences, BMI, and infertility duration were similar among the groups. Baseline clinical characteristics of the study participants are listed in Table 1.

**Table 1:** Baseline clinical characteristics of the study participants * Significance at p-value <0.05 n: number of participants, BMI: basal metabolic index, SD: standard deviation

Parameter	*Lactobacillus*-dominant (n = 68) mean ± SD	Non-*Lactobacillus*-dominant (n = 32) mean ± SD	Test used	Test value	Significance (p-value)
Mean age (years)	29.8 ± 4.2	30.2 ± 4.8	Independent t-test	t = 0.49	0.624
BMI (kg/m²)	24.1 ± 3.5	24.8 ± 3.7	Independent t-test	t = -0.73	0.468
Duration of infertility (years)	3.5 ± 1.2	3.7 ± 1.1	Independent t-test	t = -0.71	0.479

The average age was 29.8 ± 4.2 and 30.2 ± 4.8 years in the *Lactobacillus*-dominant and non-dominant groups, respectively (p = 0.624). BMI and duration of infertility were also found to be similar, with no statistically significant results (p = 0.468 and p = 0.479, respectively). These well-matched groups suggest a valid comparison of pregnancy outcomes. Table 2 demonstrates the clinical pregnancy rates by microbiome profiling.

**Table 2:** Clinical pregnancy rates by microbiome profile * Significance at p-value <0.05 n: number of participants, %: percentage

Outcome	*Lactobacillus*-dominant (n = 68)	Non-*Lactobacillus*-dominant (n = 32)	Test used	χ² value	Significance (p-value)
Clinical pregnancy rate (%)	26 (38.2%)	4 (12.5%)	Chi-square test	7.14	0.008*

Women with *Lactobacillus*-dominant profiles had significantly higher rates (26 (38.2%) of clinical pregnancy compared to non-*Lactobacillus* dominants 4 (12.5%), p = 0.008), implying that the prevalence of *Lactobacillus* is associated with increased IUI success. Table 3 summarizes the clinical pregnancy predictors after IUI treatment.

**Table 3:** Analysis showing predictors of clinical pregnancy following IUI * Significance at p-value <0.05 BMI: basal metabolic index, OR: odds ratio, CI: confidence interval, IUI: intrauterine insemination

Predictor	Adjusted OR	95% CI	Test used	Test value	Significance (p-value)
*Lactobacillus*-dominant profile	3.85	1.28-11.58	Logistic regression	5.77	0.016*
Age (per year increase)	0.97	0.89-1.06	Logistic regression	0.43	0.513
BMI (per unit increase)	0.95	0.83-1.09	Logistic regression	0.57	0.451
Duration of infertility (per year)	0.89	0.69-1.15	Logistic regression	0.78	0.378

Logistic regression analysis also confirmed that *Lactobacillus* dominance was an independent factor that positively predicted IUI success, with an adjusted OR of 3.85 (95% CI, 1.28-11.58; p = 0.016). Age, BMI, and duration of infertility were other factors that were not significant predictors of pregnancy outcomes. It suggests that the composition of the vaginal microbiome is a better predictor of IUI outcome compared to age, BMI, or duration of infertility.

## Discussion

This study aimed to determine the role of vaginal microbiome composition, especially the dominance of *Lactobacillus*, on the outcome of clinical pregnancy in women undergoing IUI treatment. The findings of this study support that the *Lactobacillus*-dominated microbiota plays a crucial role in successful implantation and clinical pregnancy. Microbiome profiling outcomes revealed that women with a *Lactobacillus*-dominant vaginal microbiome had increased clinical pregnancy rates after IUI. This finding aligns with the research reports that found protective effects of *Lactobacillus* crispatus against changes in vaginal pH, promoting sperm survival and inhibiting the interference of pathogens in conception [11,12]. It was also suggested in another study that *L. iners*, which belongs to the genus *Lactobacillus*, is less stable and often associated with adverse outcomes in reproduction than other types [13].

Microbial analysis also showed that non-*Lactobacillus*-dominant women had higher abundances of anaerobic vaginal microbiota, such as Gardnerella and Atopobium, that have been associated with chronic inflammation and poor implantation rates. This is consistent with studies demonstrating that the microbes implicated in bacterial vaginosis increase the concentration of local cytokines, negatively affecting embryo receptivity [14]. In a survey on vaginal and endometrial flora, the coordinated advantage of *Lactobacillus* colonization in both sites was highlighted [15].

The study showed that non-*Lactobacillus* patients used fewer probiotics, experienced a longer interval of infertility, and had increased subclinical infection rates. These observations confirm the previous study that microbial changes through probiotic supplementation may restore eubiosis and improve conception outcomes [16]. A recent randomized trial, however, indicated no significant difference in pregnancy outcomes when using general probiotics, which suggests that specific strains need to be further analyzed [17]. In contrast, clinical trials with targeted *L. crispatus* have supported improved live birth rates with ART procedures [18]. These findings support the possibility of microbiome assessment as a predictive and therapeutic outcome in reproductive medicine.

This study also has limitations related to its observational design, non-probability sampling, lack of species-level differentiation, and restricted causal inference, as well as the possibility of confounding factors, including diet, hygiene, sexual activity, and unreported use of probiotics and antibiotics. Future studies should conduct randomized controlled trials to assess species-specific probiotics and consider diverse populations with extended follow-up to determine continued reproductive benefits.

## Conclusions

This study demonstrated that the *Lactobacillus*-dominant vaginal microbiome group had significantly higher clinical pregnancy rates after IUI treatment cycles compared to non-*Lactobacillus* profiles. The microbial composition, specifically the presence of *L. crispatus*, indicated a favorable and stable vaginal tissue environment, promoting sperm survival and implantation.

Consistent with the aim of the research, a significant relationship between the microbiome and the clinical pregnancy in IUI cycles was identified. It implies that pretreatment microbial testing can be used to predict women at risk of poor treatment outcomes and inform personalized treatment plans.
